# Determination of levofloxacin, norfloxacin, and moxifloxacin in pharmaceutical dosage form or individually using derivative UV spectrophotometry

**DOI:** 10.1186/s13065-024-01193-4

**Published:** 2024-06-14

**Authors:** K h. Elgendy, M. Zaky, Alaa Eldin mohamed Mahmoud altorky, S. Fadel

**Affiliations:** Chemistry Department, Faculty of Science, Zagazig University, Zagazig, Egypt

**Keywords:** Levofloxacin, Norfloxacin, Moxifloxacin, Validation, Simultaneous estimation, Derivative Uv spectrophotometry

## Abstract

**Purpose:**

In this study, first, second, third, and fourth-order derivative spectrophotometric methods utilizing the peak—zero (P—O) and peak-peak (P—P) techniques of measurement were developed for the determination of levofloxacin, norfloxacin, and moxifloxacin. These methods were applied to their combined pharmaceutical dosage form or individually for levofloxacin, norfloxacin, and moxifloxacin.

**Methods:**

Linearity was established in the concentration range of 2–20 µg/mL. The procedures are simple, quick, and precise. The developed methods are sensitive, accurate, and cost-effective, demonstrating excellent correlation coefficients (R2 = 0.9998) and mean recovery values ranging from 99.20% to 100.08%, indicating a high level of precision.

**Results:**

The developed approach was effectively employed to determine the levofloxacin, norfloxacin, and moxifloxacin content in commercially available pharmaceutical dosages.

**Conclusions:**

Statistical analysis and recovery tests confirmed the method's linearity and accuracy. The results suggest that this method can be utilized for routine analysis in both bulk and commercial formulations. The simplicity, accuracy, and cost-effectiveness of the developed methods make them valuable for pharmaceutical analysis.

**Supplementary Information:**

The online version contains supplementary material available at 10.1186/s13065-024-01193-4.

## Introduction

**Fig. 1:**
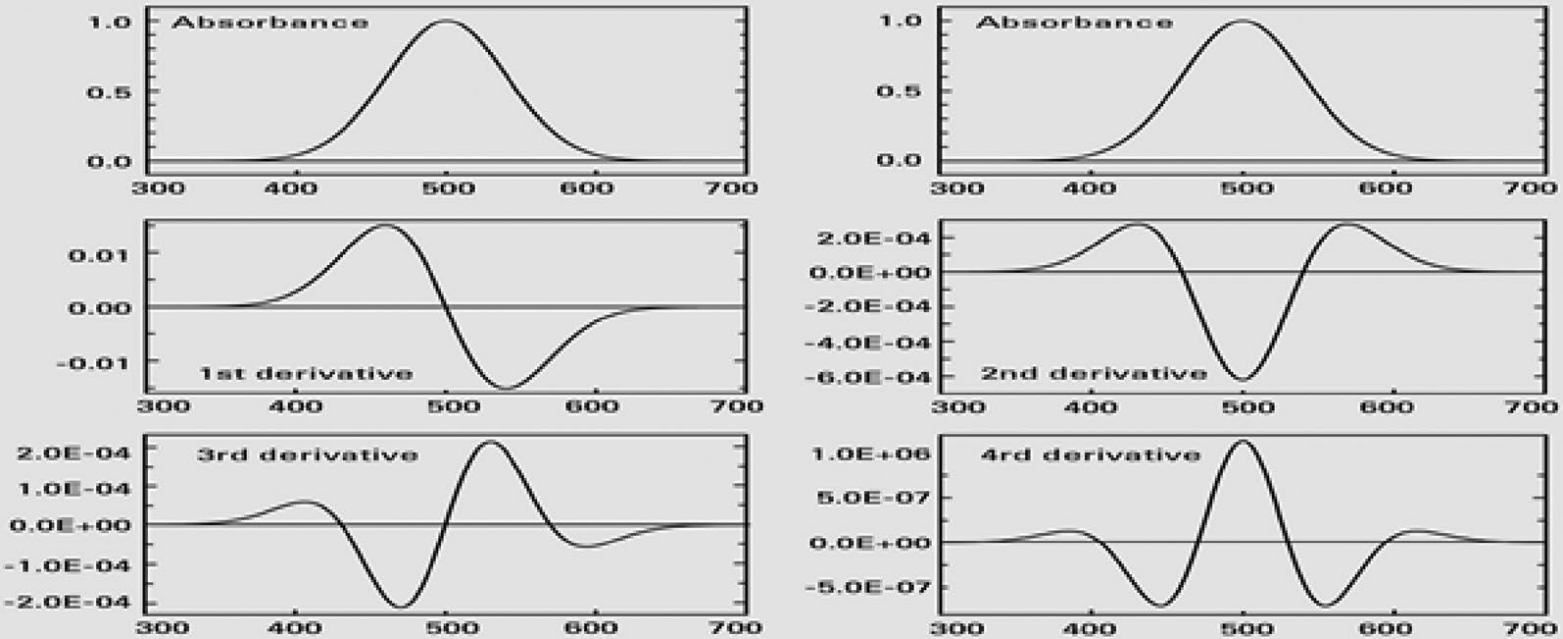
Absorbance and derivative spectra of a Gaussian band

Derived UV-spectrophotometry is a method that provides both qualitative and quantitative information from spectra in disputable bands. It involves using the first or higher derivatives of absorbance concerning wavelength for analysis purposes [1]. Derivative spectroscopy was first introduced in the 1950s and demonstrated various advantages; however, it received limited attention due to the challenges of generating derivative spectra with early UV–Visible spectrophotometers. [2] The technique gained traction with the advent of microcomputers in the late 1970s. These computers allowed for the quick, easy, and reproducible generation of derivative spectra using mathematical methods. This technological advancement significantly increased the application of the derivative technique. IN this application note, we will delve into the mathematics and generation methods of derivative spectroscopy in a concise manner. Computer-generated examples will be used to illustrate the features and applications of this technique (see Fig. 1) [20].

### Derived UV-spectrophotometry: application and methods overview

Derived UV-spectrophotometry, employing first to higher-order derivatives, provides both qualitative and quantitative information from spectra. The distinguishing feature of second-order derivatives is a negative band with a minimum at the same wavelength as the maximum on the zero-order band, along with positive satellite bands. Fourth-order derivatives display a positive band. Even-order derivatives exhibit a strong negative or positive band with a minimum or maximum at the same wavelength as the absorbance band’s maximum. The number of observed bands is one more than the derivative order [3–5]. Second-order Derivative: Negative band at the same wavelength as the zero-order maximum, with positive satellite bands. Fourth-order Derivative: Positive band. Even-order Derivatives: Strong negative or positive band with a minimum or maximum at the same wavelength as the absorbance band's maximum [6].

## Methods of derivative spectra generation

### Optical and electronic techniques

Wavelength Modulation: Involves rapid modulation of incident light wavelength over a narrow range. Suitable for generating first and second derivatives. Dual Wavelength Spectrophotometer: Produces first-derivative spectra by scanning with each monochromatic wavelength separated by a small constant difference.

### Mathematical techniques

Easily calculated and recalculated with different parameters. Smoothing techniques can enhance signal-to-noise ratio. The determination by the “peak—zero” (P—O) and “peak—peak” (P–P) techniques involves analyzing the derivative spectra to obtain specific information about the drugs being studied. Here's a more detailed explanation for both techniques:

### Peak—Zero (P—O) Technique

In the "peak—zero" technique, the analysis is based on measuring the amplitude from the maximum to the zero line or from the minimum to the zero line of the derivative spectra. The derivative spectra represent the rate of change of absorbance with respect to wavelength. Peaks or valleys in the derivative spectra correspond to specific features in the original absorbance spectrum. The "peak—zero" technique involves measuring the amplitude of a particular peak or valley in the derivative spectrum from its highest or lowest point to the zero line. This amplitude can be related to the concentration of the substance being analyzed, and the technique is used to determine the presence and concentration of specific compounds.

### Peak—Peak (P-P) Technique

In the "peak—peak" technique, the analysis is based on measuring the amplitude from one peak to another peak in the derivative spectrum. Similar to the “peak—zero” technique, the derivative spectrum is examined for peaks and valleys, and the amplitude is measured between two specific peaks. This amplitude can be used for quantification and is related to the concentration of the analyzed substance. The “peak—peak” technique provides an alternative way to extract information from the derivative spectra, allowing for a comprehensive analysis of the features present.

Both techniques essentially use the characteristics of the derivative spectra to quantify the concentration of substances. By measuring the amplitudes in different ways, analysts can choose the most suitable approach based on the specific features of the derivative spectra and the compounds being studied. These techniques are part of the broader field of derivative spectrophotometry, offering advantages in terms of sensitivity and selectivity in the determination of drugs and other chemical substances.

### Fluoroquinolones and antibacterial chemotherapy

Fluoroquinolones, a class of antibiotics structurally similar to nalidixic acid, exhibit enhanced antibacterial activity. Improved potency, broader antibacterial spectrum, and favorable pharmacokinetic properties contribute to their clinical significance. Widely distributed in the body, fluoroquinolones are effective against various infections, including urinary, respiratory, gastrointestinal, skin, soft tissue, bone, and sexually transmitted infections. The ease of oral therapy adds to their advantages.

### Quinolones and fluoroquinolones

Quinolones, structurally similar to nalidixic acid, achieve bactericidal action by blocking bacterial DNA gyrase. Early quinolones had limitations, including a narrow spectrum, low potency, high resistance rates, low serum concentrations, and short half-lives. Fluoroquinolones overcome these limitations with a broader antibacterial range, increased potency, low resistance rates, high oral bioavailability, significant tissue penetration, and extended elimination half-lives. Potential side effects include gastrointestinal disturbances, rashes, central nervous system stimulation, cartilage damage, eye toxicity, teratogenicity, and spermatogenesis impairment [7, 8]. Newer Fluoroquinolones characterized by broad-spectrum bactericidal action, excellent oral bioavailability, good tissue penetration, and favorable safety and tolerability. Classified into four generations based on antibacterial spectrum and therapeutic indications. The extended antibacterial spectrum of the most recent fluoroquinolones allows for a diverse range of applications. Spectroscopic Analysis of Levofloxacin, Norfloxacin, and Moxifloxacin, antibiotics used against various bacterial species, were studied using fluorescence and UV–visible absorption spectroscopy. Solvent polarity and drug concentration influenced fluorescence quantum yields, lifespan, and non-radiative decay. Fluorescence quenching techniques revealed binding mechanisms with caffeine, indicating ground state complexes and contributions from electrostatics, hydrogen, and Van der Waals forces [9–12].

Recently, UV–Visible spectroscopy and density functional theory (DFT) techniques, our investigation delved into the development of charge-transfer (CT) complexes. Our focus centered on exploring the interaction of iodine with aniline and its derivatives in CCl4 across varying temperatures (293.15–308.15 K). The calculation of formation constants (KCT) using the Benesi-Hildebrand plot revealed the influence of temperature changes and donor molecule structure on KCT values. Both experimental and DFT UV spectra uncovered an additional CT band, with the elongation of I2 acceptor bonds facilitating the observation of the interaction between I2 and aniline derivatives [13]. Introduced an innovative titrimetric approach (method A) for the precise determination of Ciprofloxacin Hydrochloride (CIP-HCl). This approach involved the in situ bromination of CIP-HCl through the interaction of acid with the bromate-bromide combination. Additionally, a UV-Spectroscopic analysis (method A), as per previous literature, demonstrated adherence to Beer's law within the concentration range of 3.5–11.5 g mL^−1^. Titrimetric analysis extended the determination range to 5.0–70.0 mg CIP-HCl. Comparative analysis of accuracy and precision with the reference method (method B) showcased negligible differences in results. Method A successfully determined three distinct dosage forms of CIP-HCl [14]. A novel, rapid, specific, and economical UV spectrophotometric method was established for the quantification of levofloxacin in both pure form and pharmaceutical formulations. This technique employs a distinct solvent mixture (water: methanol: acetonitrile) and measures absorbance at a pre-determined wavelength (292 nm). It was demonstrated to be linear across a broad range of levofloxacin concentrations (1.0–12.0 mg/mL), exhibiting a commendable correlation coefficient (R^2^ = 0.9998) and exceptional mean recovery (99.00–100.07%). [15, 16]. Study investigated the application of highly crystalline TiO_2_ nanoparticles in the photocatalytic degradation of levofloxacin. Synthesized through a sol–gel method, the TiO_2_ nanoparticles underwent comprehensive assessment for morphological, structural, compositional, thermal, and optical characteristics. The in-depth research highlighted the nanoparticles' high density, exceptional crystallinity, and favorable optical characteristics, contributing to efficient photocatalytic degradation [17]. Employing a straightforward hydrothermal and sol–gel synthesis, we created carbon dots (C-dots), titanium dioxide (TiO_2_) quantum dots, and TiO_2_/C-dots. Thorough characterization encompassing crystallinity, structure, morphology, thermal stability, and optical properties confirmed the great quality of the produced photo catalysts. The nanocomposites displayed an average size of 12 nm, with C-dots uniformly dispersed across TiO_2_ quantum dots [18]. Addressing contemporary environmental challenges, our emphasis centered on the need for photocatalytic materials with suitable structural and morphological architectures. The creation of Z-scheme hetero junctions emerged as an ideal strategy to overcome the limitations associated with single-component or conventional heterogeneous catalysts. Our discussion highlighted the diverse applications of these materials, particularly in the effective removal of organic compounds from wastewater [19]. Presenting a simple, quick, and cost-effective technique, we detailed the preparation of MIL-100(Fe) using a sealed autoclave and a solid-state reaction. Extensive characterization using PXRD, FTIR, SEM, EDX, TGA, BET surface area, and zeta potential analysis provided insights into the properties of the as-prepared MIL-100(Fe). The resulting MIL-100(Fe) effectively served as an adsorbent for the extraction of the antibiotic levofloxacin from the aqueous phase [20]. More research technique were reviewed [21–37].

## Materials and methods

### Devices

UV–Visible double-beam spectrophotometric, Shimadzu 1800 Origin, Kyoto, Japan, was employed. Absorbance measurements were conducted using Uv. Probe 2.34, Model Shimadzu Origin Kyoto Japan, with matched quartz cells. Additional equipment included a laboratory oven-sonorous, a sensitive electronic balance, an ultrasonic device (ultrasonicator)-sonorex, a water bath (Karl K olb), and a hot plate with magnetic stirrer (LMS-1003, Daihan Lab Tech) from Germany. Precision measurements were facilitated by micro pipettes (1–10 µl and 100 µl) originating from Switzerland. Computational aspects were handled on a Dell computer from Germany and China.

### Chemical materials

Levofloxacin, Norfloxacin, and Moxifloxacin were generously supplied by Arti (India). Eipico Company, located in the 10th of Ramadan City, Egypt, provided pharmaceutical-grade excipients, including Citric acid, Magnesium stearate, Vinyl pyrrolidone, Starch, Talc, and Lactose. Deionized water utilized in the analysis underwent preparation through reverse osmosis and filtration via a 0.45 μm Millipore filter (Millipore Company, USA).

### Market sample

Tablet forms of Levofloxacin, Norfloxacin, and Moxifloxacin were randomly selected from the market. The recently developed and approved method was applied to analyze sample solutions against the reference standard. Quantities of Levofloxacin, Norfloxacin, and Moxifloxacin were determined using data from the marketed products.

Levoxin (250 mg Levofloxacin) by ALAR LABORATORIES PVT LIMITED, Composition**:** Levofloxacin**:** 250 mg, and excipients**:** maize starch, Magnesium stearate, Polyethylene glycol, Povidone K30, Yellow Tartrazine, and Talc.

Epinor (400 mg Norfloxacin) by EIPICO. Composition**:** Norfloxacin**:** 400 mg Excipients**:** maize starch, Magnesium stearate, Polyethylene glycol, Povidone K90, Titanium dioxide, and Talc.

Delmoxa (400 mg Moxifloxacin) by DELTA PHARMA. Composition**:** Moxifloxacin**:** 400 mg, and excipients**:** maize starch, Magnesium stearate, Lactose, Povidone K90, Talc, and Iron oxide red.

### Solutions

Solution (1000 µg/mL): Prepared by dissolving 0.1 g of each substance in 100 ml of 0.1 mol/L HCl solution. Solution (100 µg/mL): Obtained by diluting the stock solution in methanol and further dilution in a volumetric flask to achieve a concentration of 100 µg/mL in 0.1 mol/L HCl solution (2–20 µg/mL). Additives (1000 µg/mL): Solution containing Levofloxacin, Norfloxacin, Moxifloxacin, and other additives, prepared in 100 mL of 0.1 mol/L HCl.

### Stability study

#### Solvent selection and standardization

In the preliminary trial, five different solvent compositions were considered, including distilled water, methanol, methanol: distilled water (1:1), 0.1 N HCl, and phosphate buffer (pH 7.1). After evaluation, methanol: distilled water (1:1) was chosen as the suitable medium due to its ease of sample preparation, the drug's solubility, and cost-effectiveness. The wavelength maximum for levofloxacin, norfloxacin, and moxifloxacin was identified at 287 nm,291 nm, and 294 respectively A stock solution of the levofloxacin, norfloxacin, and moxifloxacin standard were prepared with an approximate concentration of 5 μg/mL and subjected to sonication for 4 min in a sonicator bath.

#### Linearity

The response function was determined by preparing standard solutions at ten different concentration levels ranging from 2 to 20 μg/mL using UV/Vis derivative spectrophotometric methods. The limit of detection (LOD) and limit of quantitation (LOQ) were calculated based on the standard deviation of the Y-intercept and the slope of the calibration curve following the International Council for Harmonisation (ICH) guidelines [20]. The formulas used were LOD = 3.3 × (standard deviation of Y-intercept/slope of the curve) and LOQ = 10 × 2 (standard deviation of Y-intercept/slope of the curve).

#### Accuracy

A study was conducted using pre-formulated granules containing pure Levofloxacin, Norfloxacin, and Moxifloxacin, along with common excipients. The calculation was based on the label claim and the average weight of the final product. A previously established dilution pattern was followed for the granules, resulting in three concentrations 80%, 100%, and 120% of the reference solution. Three replicate samples were prepared at each concentration level, and the percent recovery at each level (n = 10) was determined.

#### Precision

By repeatedly scanning levofloxacin, norfloxacin, and moxifloxacin standard samples (n = 6) without altering the parameters of the suggested derivative spectrophotometric method, the instrumental precision, expressed as relative standard deviation (RSD), was verified. Data on intra-assay and inter-assay precision (RSD) were collected in the lab on days 1, 3, and 7 as well as on several days over the course of a week (days 1, 3, 5, and 7). The standard deviation of the measurements divided by the mean of the measurements multiplied by 100 is how the RSD percentage is computed.

#### Assay

Three marketed brands (Levoxin, Epinor and Delmoxa) were tested using this method, with levofloxacin at 287 nm, norfloxacin at 281 nm and moxifloxacin at 294 nm, using the following formula: active ingredient content (mg/ tablet). = (sample absorbance/standard absorbance) × (standard weight/sample weight) × average weight × standard intensity/100.

Samples generated for repeatability research were stored at room temperature for 24 h before undergoing testing for short-term stability the following day.

### Specificity in the presence of excipients

An analytical method’s specificity is intended to quantitatively identify the analyte as a constituent that is anticipated to exist in the sample matrix. Popular additives, such as lactose, povidone K30, magnesium stearate, and purified talc, are combined in the right proportions per test protocol and dissolved in a solvent system that matches the additive weight during sample preparation. First, the nominal concentration of the drug is combined with various additive concentrations (80–120% of the nominal concentration in the test formulation). After increasing the additive's nominal concentration to different drug concentration levels (80–120% of the test formulation's nominal concentration), absorption is assessed. To calculate how much medication is needed, perform some calculations. The response of commercially available products, standard of levofloxacin, norfloxacin, and moxifloxacin, and additives under stress conditions was compared with the response of the same solutions under stress conditions in order to determine the stability-indicating properties of the developed UV/visible method. This analysis is a component of a study on forced degradation.

### Forced degradation study

The drug material and one of the three commercially available drug products were both forced to degrade under various stress conditions in this study. These conditions included neutral, acidic, and base hydrolysis as well as oxidative, photolytic, and thermolytic stress. Specifically, the drug substance and drug product degradation was carried out in the solid state. The drug substance or drug product is dissolved in distilled water, aqueous HCl/NaOH/H_2_O_2_, or a solvent until a concentration of 50 μg/mL is reached to be able to start a degradation study. Subsequently, these solutions were diluted using a methanol: distilled water (1:1) solvent to a concentration of roughly 5 μg/mL. The degradation was then observed by performing a UV/visible spectrophotometric, a spectral scan in the 220–350 nm range, and measuring the absorbance on various days. Hydrolysis tests were performed to evaluate the impact of different conditions on the drug substance and drug product. For duration of seven days, neutral hydrolysis using distilled water was conducted at room temperature and 60 ℃. Using 0.1 N HCl, acid hydrolysis was carried out for seven days at 60 ℃ and room temperature. In contrast, base hydrolysis used 0.1 N NaOH solutions at 60 ℃ and room temperature, but only for two days. Sample solutions of the drug substance and drug product were exposed to 3% H_2_O_2_ at room temperature and 60 ℃ for a week in order to assess oxidative stress. Over 14 days, photolytic stress experiments were conducted, and both light and dark conditions were observed. The drug substance and drug product were subjected to controlled oven temperatures—room temperature and 70 ℃, specifically—for 14 days to induce thermal stress. It is significant to remember that all samples under stress were given placebo preparations.

### Determination of levofloxacin, norfloxacin, and moxifloxacin in pharmaceutical dosage

Weighed and milled were ten tablets of Levonex (0.250 g of levofloxacin), Epinor (0.400 g of norfloxacin), and Deltamox (0.400 g of moxifloxacin). Each tested drug’s 50 mg of homogenized powder was carefully weighed and added to a 50 mL volumetric flask along with 20 mL of 0.1 mol/L HCl. 0.1 mol/L HCl was used to dilute the volumes to 50 ml after the combinations were shaken for 20 min. The resulting solutions were then filtered. Then, using 0.1 mol/L HCl, 1 mL of each clear solution was diluted to 10 mL. These solutions' volumes—0.5 mL, 0.5 mL, and 1.0 mL for the levofloxacin, norfloxacin, and moxifloxacin solutions, respectively—were put into 10 mL volumetric flasks and diluted with 0.1 mol/l HCl until the proper volume was reached. For these solutions, derivative spectra at the first, second, third, and fourth orders were noted. The concentrations of Levofloxacin, Norfloxacin, and Moxifloxacin in the studied sample solutions were determined by interpolating the corresponding calibration curves, and the amplitudes of the minimum and maximum were graphically measured.

## Results

### Method development and optimization

Levofloxacin, Norfloxacin, and Moxifloxacin demonstrated high solubility in 0.1 mol HCl.The optimized solvent composition was water (5): methanol (5) for favorable UV analysis. The absorption spectra were scanned in the range of 220–350 nm, revealing maximum absorption wavelengths (λ max) for Levofloxacin at 287 nm, Norfloxacin at 291 nm, and Moxifloxacin at 294 nm.

### Method validation

#### Linearity and range

The calibration curve exhibited a linear correlation coefficient (R2) exceeding 0.99. Calibration curves for derivative spectra of each drug were constructed, showing significant clarity in the graphs.

Plotting the graphically measured (mm) amplitudes of the first-, second-, third-, and fourth-order derivative spectra against the corresponding concentrations of the drugs under investigation allowed for the construction of calibration curves. The first, second, third, and fourth-order derivative spectra of the levofloxacin standard solution in 0.1 mol HCI are displayed in Figure 3. They were recorded at concentrations of 2.0 to 20.0 µg mL of levofloxacin, within the wavelength range of 220–350 nm. The first, second, third, and fourth-order derivative spectra of the norfloxacin standard solution in 0.1 mol HCI are displayed in Figure 4. The spectra were recorded at concentrations of 2.0 to 20.0 µg mL of norfloxacin, within the wavelength range of 220–350 nm. The first, second, third, and fourth-order derivative spectra of the moxifloxacin standard solution in 0.1 mol HCI are displayed in Figure 5. The spectra were recorded at concentrations of 2.0 to 20.0 µg mL of moxifloxacin, within the wavelength range of 220–350 nm. Tables l0, 12, and 14 present the linear equations derived from the regression analysis of the ciprofloxacin hydrochloride, norfloxacin, and ofloxacin data, respectively. The data on the determination of the studied fluoroquinolones in tablets, along with a statistical analysis of the outcomes, are displayed in Tables 11, 13, and 15.

#### Limit of detection and limit of quantification

**Fig. 2:**
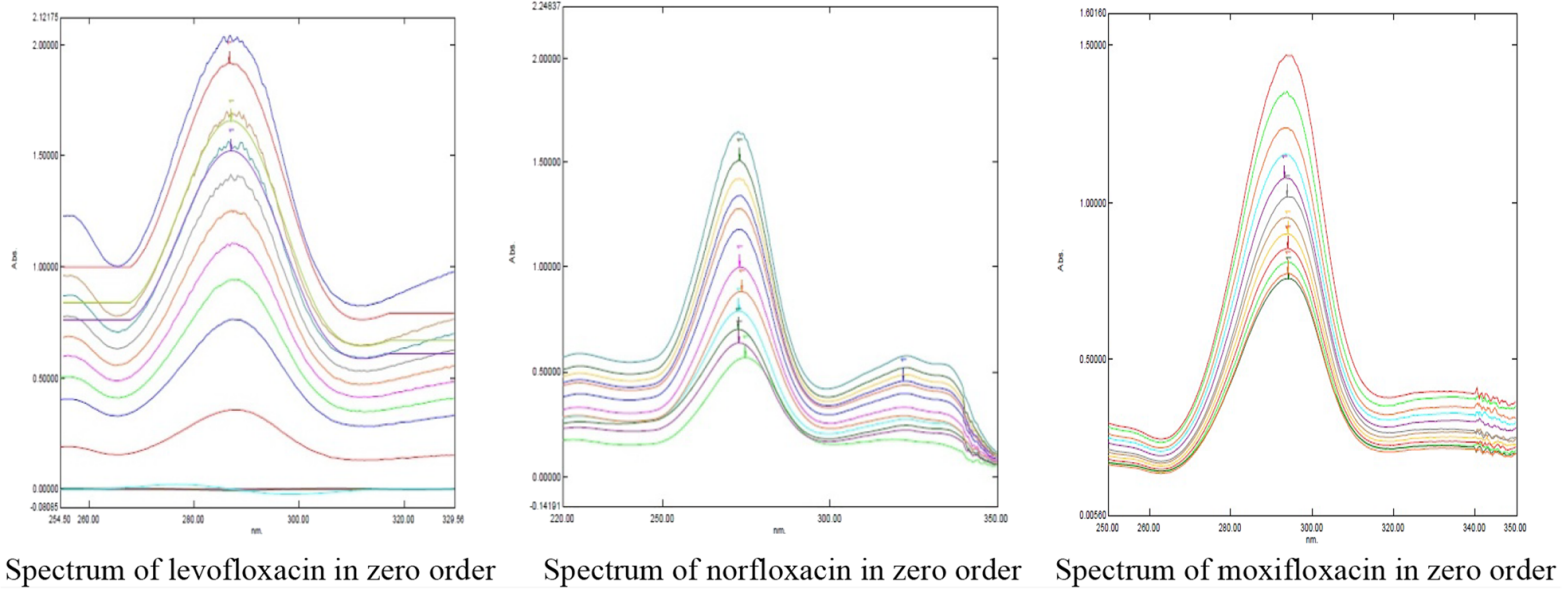
Spectrums of levofloxacin, norfloxacin, and moxifloxacin in zero order

LOD and LOQ values, calculated from linearity studies, indicated high sensitivity of the proposed method in the range of 2-20 µg/mL for Levofloxacin, Norfloxacin, and Moxifloxacin. Are displayed in Figure 2

#### Stability

Stability study results (Table 1) indicated that the samples were stable for 24 h (short-term) at various concentrations, affirming the robustness of the proposed method.

**Table 1:** Short-term Stability

Drugs	Concentration declared (mg/mL)	Concentration found (mean ± SD)	RSD (%)	Average potency (%)
Levofloxacin	2	1.96 ± 0.05	0.295	98.19
	4	3.95 ± 0.05	0.146	98.80
	6	5.97 ± 0.05	0.097	99.41
Norfloxacin	2	1.94 ± 0.05	0.295	98.20
	4	3.90 ± 0.05	0.265	99.90
	6	5.93 ± 0.05	0.098	100.05
Moxifloxacin	2	1.95 ± 0.05	0.295	98.19
	4	3.96 ± 0.05	0.150	99.50
	6	5.98 ± 0.05	0.255	98.90

For all three medications at various concentrations (2, 4, and 6 mg/mL), the values of the concentration found generally agree with the claimed concentration. All drug potencies vary from 98.19% to 100.05% on average, which shows that there is good agreement between the declared and actual concentrations. The measurements are consistent and show little fluctuation around the mean when the RSD values are low.

#### Accuracy/recovery

Results within the range of 99.20–100.08% ensure an accurate method as shown in (Table 2).

**Table 2:** Recovery/accuracy for three different concentrations of Levofloxacin, Norfloxacin, and Moxifloxacin

Dosage form	Label claim	Amount added (%)	Recovery (%)
levofloxacin	0.250 mg	80	99.80
		100	99.45
		120	99.60
Norfloxacin	0.400 mg	80	100.05
		100	99.70
		120	100.01
Moxifloxacin	0.400 mg	80	99.5
		100	100.08
		120	99.20

#### Precision

The repeatability and intra-day assay precision (RSD) was < 1% and the inter-day assay precision was < 2% revealed the proposed method for analyzing Levofloxacin is both precise and accurate (Table 3).

**Table 3:** Precision Study levofloxacin

Sample No	% Assay	Intra-day assay			Inter-day assay			
	(repeatability)	1st h	3rd h	8th h	1st day	3rd day	5th day	7th day
1	100.8	99.25	100.25	99.85	101.52	100.58	99.52	100.25
2	101.85	99.65	100.35	99.65	98	100.52	100.2	100.6
3	101.65	99.4	100.45	100.2	99.5	99	99.8	100.2
4	101.6	99.5	100.3	99.68	98.8	102.5	102.5	99.5
5	101.55	99.58	100.58	99.5	100.25	101.5	99.8	99.6
6	101.4	99.65	100.45	99.85	101.8	99.85	101.5	99.54
Mean ± SD	101.74 ± 0.36	99.54 ± 0.16	100.4 ± 0.085	99.76 ± 0.25	99.87 ± 1.50	100.55 ± 1.43	100.0 ± 1.13	99.9 ± 0.461
% RSD	0.361593	0.168325	0.085391	0.252521	1.506906	1.43373638	1.358958	0.460751

The repeatability and intra-day assay precision (RSD) was < 1% and the inter-day assay precision was < 2% revealed the proposed method for analyzing norfloxacin n is both precise and accurate (Table 4).

**Table 4:** Precision Study norfloxacin

Sample No	% Assay		Intra-day assay			Inter-day assay			
	(repeatability)		1st h	3rd h	8th h	1st day	3rd day	5th day	7th day
1	100.07		99.58	100.25	99.5	100.2	100.2	98.2	100.52
2	100.5		99.5	100.39	99.5	99.8	99.8	100.5	100.2
3	101.5		100.25	101.25	101.2	99.2	99.6	100.9	100.25
4	100.25		101.02	100.2	99.5	100.8	101.5	100.8	98.9
5	100.25		99.6	99.85	99.35	101.25	101.8	99.9	99.6
6	100.2		99.58	99.8	99.7	100.18	100.2	100.9	99.2
Mean ± SD	100.25 ± 0.52		99.59 ± 0.60	100.2 ± 0.52	99.5 ± 0.69	100.19 ± 0.72	100.2 ± 9.13	100.65 ± 1.05	99.9 ± 0.646
% RSD	0.527462		0.604795	0.524404	0.698868	0.723061	0.91305349	1.050714	0.646233

The repeatability and intra-day assay precision (RSD) was < 1% and the inter-day assay precision was < 2% revealed the proposed method for analyzing moxifloxacin in n is both precise and accurate (Table 5).

**Table 5:** Precision Study moxifloxacin

Sample No	% Assay		Intra-day assay			Inter-day assay			
	(repeatability)		1st h	3^rd^ h	8th h	1st day	3rd day	5th day	7th day
1	99.5		99.5	100.2	99.5	100.6	101.25	99.5	99.5
2	100.2		99.8	100.25	99.25	99.2	99.2	99.7	99.2
3	100.6		100.2	100.5	100.6	100.5	99.4	101.25	99.6
4	101.5		100.8	101.6	99.7	100.6	99.8	100.4	100.52
5	100.3		101.6	99.75	99.2	100.7	100.5	99.25	101.2
6	99.25		99.8	99.65	99.9	101.25	101.25	98.9	100.6
Mean ± SD	100.25 ± 0.80		100.0 ± 0.78	100.2 ± 0.70	99.6 ± 0.51	100.6 ± 0.67	100.15 ± 0.90	99.6 ± 0.85	100.06 ± 0.78
% RSD	0.834666		0.561991	0.654949	0.587899	0.684957	0.92590046	0.792017	0.5692978

The ability of a method to measure the analytical response accurately in the presence of all possible components is referred to as specificity. Furthermore, confirming the suggested method's specificity is the sample’s stress analysis. The study aimed to determine the critical variables that will affect the drug product's stability and validate the developed method's ability to indicate stability. To assess the interference of degradation products in the quantitation of levofloxacin, norfloxacin, and moxifloxacin, the specificity was determined by ICH guidelines by subjecting a sample solution and solid to accelerated degradation by acidic/alkaline/neutral hydrolytic, oxidative, photolytic, and thermal stress conditions. The reported method provides data on specificity for their estimation in the presence of degradants and formulation excipients.

#### Specificity in the presence of excipients

The specificity of the method was confirmed by comparing spectra of placebo granules and degradation products with that of accurate samples.

### Study effect of additives

First derivative of peak absorbance measurement at wavelength (287) nm, second derivative of peak absorbance measurement at wavelength (287) nm, third derivative of peak absorbance measurement at wavelength (287) nm, and fourth derivative of peak absorbance measurement at wavelength (287) nm were used to determine the effect of specific pharmaceutical additives on levofloxacin. The estimation of levofloxacin at10 μg/mL does not exhibit any additive effect, as indicated by the fourth derivative of the peak absorbance measurement. The outcomes are displayed in Table 6.

**Table 6:** The suggested method's effect of additives (100 µg/mL) on the drug levofloxacin determination

Additives	First D1		Rec %	Second D2		Rec %	Third D3		Rec %	Fourth D4		Rec %
	λ(287)nm			λ(287)nm			λ(287)nm			λ(287)nm		
	Taken (µg/mL)	Found (µg/mL)		Taken (µg/mL)	Found (µg/mL)		Taken (µg/mL)	Found (µg/mL)		Taken (µg/mL)	Found (µg/mL)	
Talc	10	9.9	99	10	9.85	98.5	10	9.85	98.5	10	9.85	98.5
Citric acid	10	9.85	98.5	10	9.95	99.5	10	9.9	99	10	9.77	97.7
Starch	10	9.9	99	10	9.92	99.2	10	9.85	98.5	10	9.85	98.5
Magnesium stearate	10	9.8	98	10	9.97	99.7	10	9.98	99.8	10	9.9	99
Lactose	10	9.75	97.5	10	9.86	98.6	10	9.9	99	10	9.95	99.5
Vinyl pyrrolidone	10	9.95	99.5	10	9.77	97.7	10	9.87	98.7	10	9.8	98

First derivative of peak absorbance measurement at wavelength (291) nm, second derivative of peak absorbance measurement at wavelength (291) nm, third derivative of peak absorbance measurement at wavelength (291) nm and fourth derivative of peak absorbance measurement at wavelength (291) nm were used to determine the effect of specific pharmaceutical additives on norfloxacin. The estimation of Norfloxacin at 10 μg/mL does not exhibit any additive effect, as indicated by the fourth derivative of the peak absorbance measurement. The outcomes are displayed in Table 7.

**Table 7:** The suggested method’s effect of additives (100 µg/mL) on the drug norfloxacin determination

Additives	First D1		Rec %	Second D2		Rec %	Third D3		Rec %	Fourth D4		Rec %
	λ(287)nm			λ(287)nm			λ(287)nm			λ(287)nm		
	Taken (µg/mL)	Found (µg/mL)		Taken (µg/mL)	Found (µg/mL)		Taken (µg/mL)	Found (µg/mL)		Taken (µg/mL)	Found (µg/mL)	
Talc	10	9.80	98	10	9.95	99.5	10	9.90	99.0	10	9.75	97.5
Citric acid	10	9.9	99	10	9.90	99.0	10	9.85	98.5	10	9.75	97.5
Starch	10	9.8	98	10	9.9	99.0	10	9.90	99	10	9.98	99.8
Magnesium stearate	10	9.85	98.5	10	9.8	98	10	9.95	99.5	10	9.95	99.5
Lactose	10	9.85	98.5	10	9.85	98.5	10	9.85	98.5	10	9.9	99
Vinyl pyrrolidone	10	9.9	99	10	9.95	99.5	10	9.85	98.5	10	9.7	97

First derivative of peak absorbance measurement at wavelength (294) nm, second derivative of peak absorbance measurement at wavelength (294) nm, third derivative of peak absorbance measurement at wavelength (294) nm and fourth derivative of peak absorbance measurement at wavelength (294) nm were used to determine the effect of specific pharmaceutical additives on moxifloxacin. Estimation of moxifloxacin at 10 μg/mL does not exhibit any additive effect, as indicated by the fourth derivative of the peak absorbance measurement. The outcomes are displayed in Table 8.

**Table 8:** The suggested method’s effect of additives (100 µg/ml) on the drug moxifloxacin determination

Additives	First D1		Rec %	Second D2		Rec %	Third D3		Rec %	Fourth D4		Rec %
	λ(287)nm			λ(287)nm			λ(287)nm			λ(287)nm		
	Taken (µg/mL)	Found (µg/mL)		Taken (µg/mL)	Found (µg/mL)		Taken (µg/mL)	Found (µg/mL)		Taken (µg/mL)	Found (µg/mL)	
Talc	10	9.95	99.5	10	98.5	98.5	10	9.75	97.5	10	9.88	98.8
Citric acid	10	9.9	99	10	9.8	98.0	10	9.8	98	10	9.97	99.7
Starch	10	9.85	98.5	10	9.82	98.2	10	9.95	99.5	10	9.95	99.5
Magnesium stearate	10	9.75	97.5	10	9.92	99.2	10	9.88	98.8	10	9.95	99.5
Lactose	10	9.95	99.5	10	9.78	97.8	10	9.78	97.8	10	9.8	98.0
Vinyl pyrrolidone	10	9.95	99.5	10	9.77	97.7	10	9.87	98.7	10	9.8	98

#### Forced degradation study

The stress test findings for three antibiotics—levofloxacin, norfloxacin, and moxifloxacin—are displayed in the table. In order to observe how drugs degrade, the test subjects them to more severe conditions than they usually encounter. The most stable appears to be levofloxacin. In most cases, levofloxacin degrades more slowly than norfloxacin. Similar to norfloxacin, moxifloxacin breaks down a little bit more slowly (Table 9).

**Table 9:** Forced degradation study of levofloxacin, norfloxacin, and moxifloxacin

			Levofloxacin		Norfloxacin		Moxifloxacin	
			% Assay of Standard	% Assay of product	% Assay of standard	% Assay of product		
							% Assay of standard	% Assay of product
Hydrolysis	Neutral hydrolysis at RT*	7th	90.25	103.45	89.98	104.54	99.5	103.54
	Neutral hydrolysis at 60°	7th	125.93	100.5	135.93	101.78	105.93	101.78
	Acid hydrolysis at RT*	7th	89.69	98.5	89.69	94.04	99.69	94.04
	Acid hydrolysis at 60°	5th	99.59	85.58	93.59	83.37	96.59	83.37
		6th	112.93	142.35	112.93	142.35	112.93	142.35
	Base hydrolysis at RT*	3rd	60.79	60.5	59.79	66.78	60.5	66.78
	Base hydrolysis at 60°	1st	27.8	82.5	37.8	72.78	55.05	72.78
Photolytic	Dark (solution state)	14th	118.64	105.68	108.64	106.68	103.6	106.68
	Light (solution state)	14th	103.48	122.28	102.48	142.28	102.48	132.28
Oxidation	30% H_2_O_2_ at RT*	7th	85.54	102.72	80.54	100.72	90.54	100.72
	30% H_2_O_2_ at 60°	7th	126.76	109.29	106.76	108.29	102.76	108.29
Thermal (solid state)	RT*	14th	106.2	99.18	102.2	99.08	102.2	99.5
	60°	14th	101.72	104.83	112.40	104.83	111.72	101.83

^*^RT Room Temperature

Figure 2 aims to illustrate the absorption characteristics of Levofloxacin, Norfloxacin, and Moxifloxacin. By comparing these spectra, can potentially. Identify and differentiate between the three compounds based on their unique peak patterns, gain insights into the structural similarities and differences between the molecules, provide a reference for further analysis using techniques derivative spectroscopy. In the “peak-peak” technique, amplitude measurements (from maximum to minimum) were conducted, while the baseline-to-peak technique involved measurements from the maximum to the zero line or from the minimum to the zero line (Additional file 1).

**Fig. 3:**
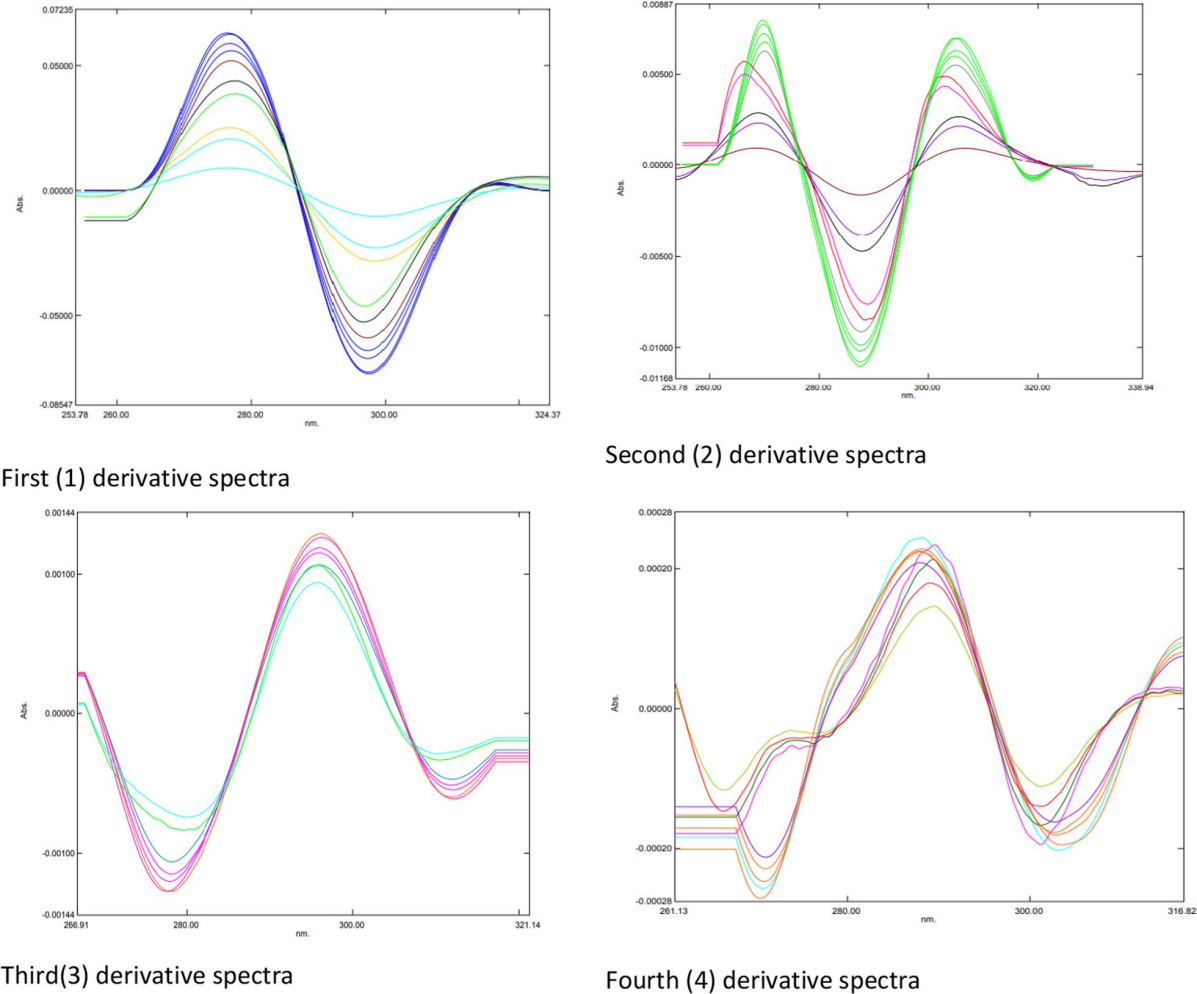
First (l) second (2), third (3) and fourth (4) derivative spectra of levofloxacin

Figure 3 presents the first (1st), second (2nd), third (3rd), and fourth (4th) derivative spectra of levofloxacin. Each spectrum exhibits distinct features compared to the original spectrum and other derivatives. The purpose of these derivative spectra is to enhance specific characteristics and potentially improve the analysis of levofloxacin.

**Fig. 4:**
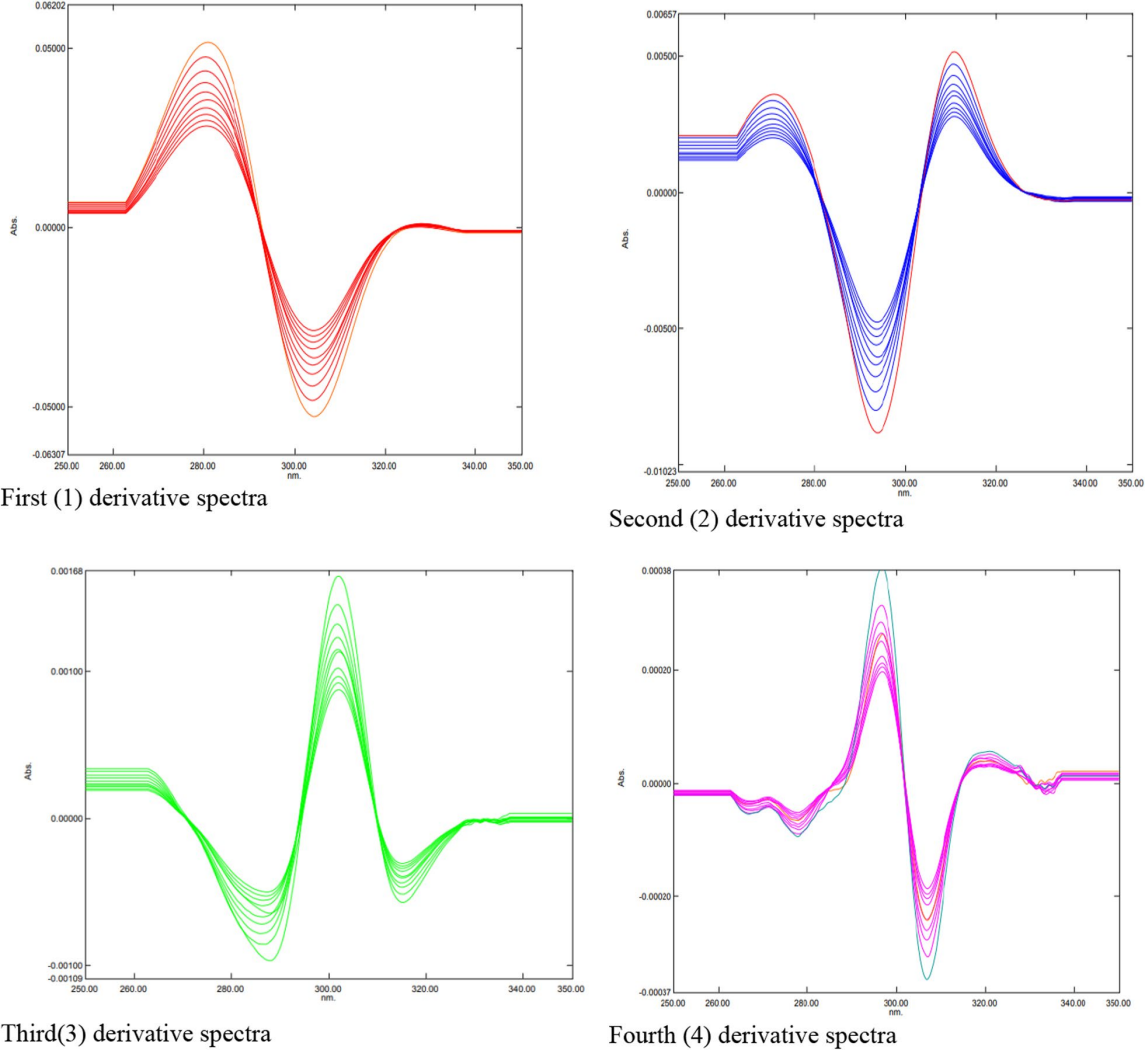
First (l) second (2), third (3) and fourth (4) derivative spectra of norfloxacin

Figure 4 presents the first (1st), second (2nd), third (3rd), and fourth (4th) derivative spectra of norfloxacin. Each spectrum exhibits distinct features compared to the original spectrum and other derivatives. The purpose of these derivative spectra is to enhance specific characteristics and potentially improve the analysis of norfloxacin.

**Fig. 5:**
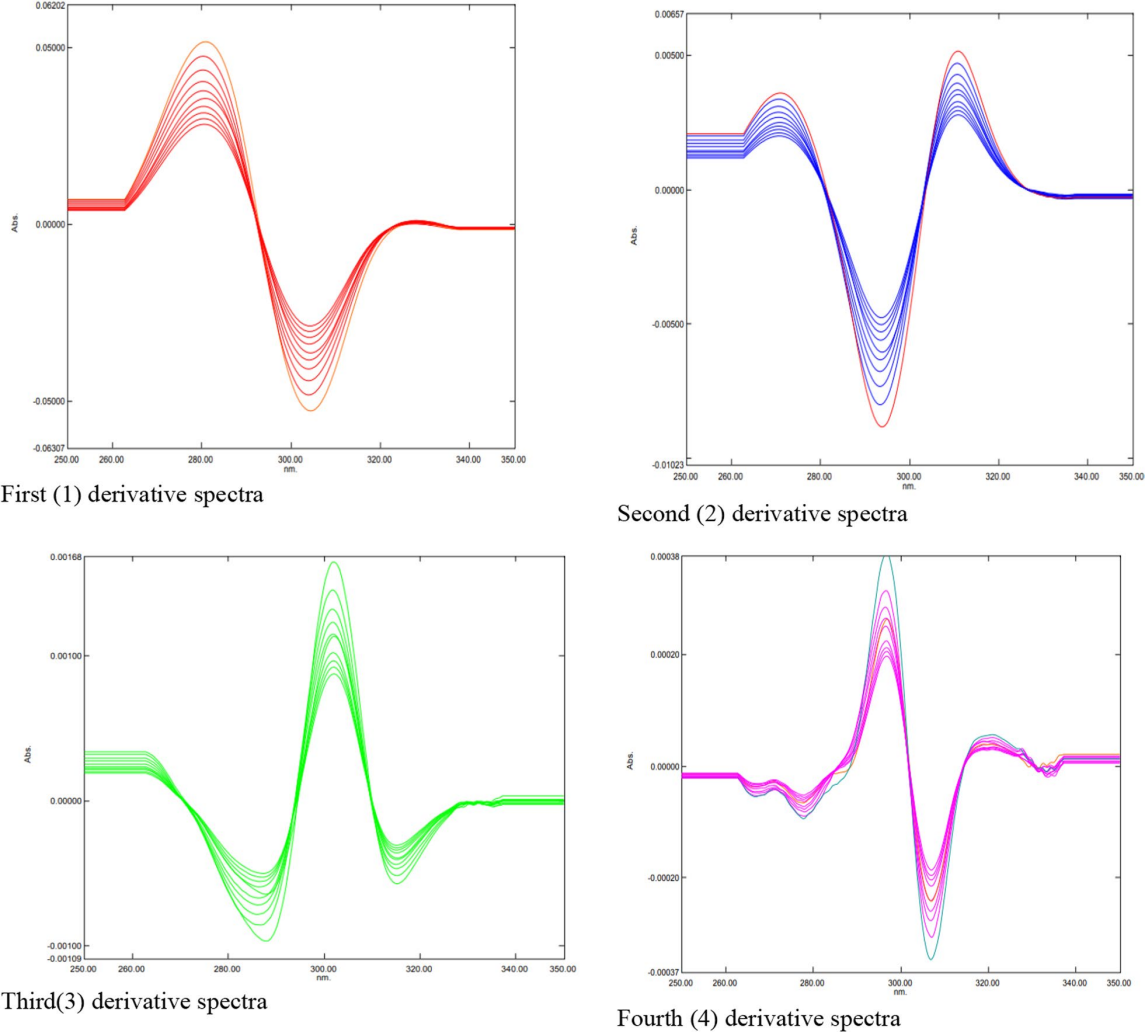
First (l) second (2), third (3) and fourth (4) derivative spectra of moxifloxacin

Figure 5 presents the first (1st), second (2nd), third (3rd), and fourth (4th) derivative spectra of moxifloxacin. Each spectrum exhibits distinct features compared to the original spectrum and other derivatives. The purpose of these derivative spectra is to enhance specific characteristics and potentially improve the analysis of moxifloxacin Additional file 1.

#### Statistical analysis

The data were meticulously subjected to statistical analysis, expressing results in terms of standard deviation (%SD) and relative standard deviation (%RSD). The calculated %RSD values, consistently below 1%, underscore the remarkably high precision achieved by these methods (refer to Tables 10, 12, 14). This statistical assessment further solidifies the reliability and reproducibility of the developed methods. The content determination of Levofloxacin, Norfloxacin, and Moxifloxacin in market products closely aligned with label claims, ranging from 98.05% to 99.47%, indicating the reliability of the proposed method. RSD values were within the range of 0.16% to 0.92% (refer to Tables 11, 13, 15).

**Table 10:** Statistical evaluation of the developed method for levofloxacin (standard solution, n = 10)

Derivative	λ (nm)	Technique	Regression equation	Correlation coefficient
D1	275	Absorbance (p-o)	y = 0.0029x + 0.0095	0.946
D1	297	Absorbance (p-o)	y = − 0.0033x–0.0112	0.934
D1	286–322	Peak(P-P)	y = − 0.0504x–0.1996	0.989
D1	257–286	Peak(P-P)	y = 0.0335x + 0.2049	0.982
D1	257–322	Peak(P-P)	y = − 0.0164x + 0.0024	0.988
D2	305	Absorbance (p-o)	y = 0.0003x + 0.0009	0.969
D2	287	Absorbance (p-o)	y = − 0.0005x–0.0022	0.970
D2	296	Absorbance (p-o)	y = 0.0004x + 0.001	0.988
D2	262–276	Peak(P-P)	y = 0.0023x + 0.0179	0.986
D2	276–297	Peak(P-P)	y = − 0.0066x–0.0117	0.976
D2	297–318	Peak(P-P)	y = 0.0041x + 0.0113	0.987
D2	262–318	Peak(P-P)	y = 0.0003x + 0.0116	0.980
D3	278	Absorbance (p-o)	y = − 0.00006x–0.00021	0.974
D3	296	Absorbance (p-o)	y = 0.00005x + 0.0003	0.985
D3	311	Absorbance (p-o)	y = − 0.00003x–0.00008	0.980
D3	267–287	Peak(P-P)	y = − 0.0007x–0.0043	0.983
D3	287–306	Peak(P-P)	y = 0.0006x + 0.005	0.979
D3	306–317	Peak(P-P)	y = − 0.0001x–0.0003	0.997
D3	267–317	Peak(P-P)	y = 0.0002x–0.0078	0.977
D4	270	Absorbance (p-o)	y = − 0.00001x–0.00008	0.986
D4	287	Absorbance (p-o)	y = 0.00001x + 0.00012	0.993
D4	304	Absorbance (p-o)	y = − 0.00001x–0.00007	0.988
D4	316	Absorbance (p-o)	y = 0.000005x–0.000004	0.993
D4	267–275	Peak(P-P)	y = − 0.00002x–0.00018	0.968
D4	275–296	Peak(P-P)	y = 0.0002x + 0.0006	0.980
D4	296–314	Peak(P-P)	y = − 0.00009x–0.00053	0.991
D4	314–319	Peak(P-P)	y = 0.00001x + 0.00004	0.980
D4	267- 319	Peak(P-P)	y = 0.00006x + 0.00029	0.973

p-o refers to point-to-origin, P-P refers to peak-to-peak

**Table 11:** Statistical analysis of the determination of levofloxacin in levoxin tablet (n = 10)

Derivative	λ (nm)	Technique	Content of Levofloxacin (g)	Standard Deviation	Variance	Correlation Coefficient	Confidence Interval (RSD)—95%
D1	275	Absorbance (p-o)	0.253	8.25*10^-3	9.36*10^-5	0.81	0.243–0.264
D1	297	Absorbance (p-o)	0.251	8.65*10^-3	7.59*10^-5	0.34	0.243–0.262
D1	286–322	Peak(P-P)	0.253	7.59*10^-3	7.22*10^-5	0.15	0.245–0.264
D1	257–286	Peak(P-P)	0.252	8.32*10^-3	8.24*10^-5	0.24	0.244–0.261
D1	257–322	Peak(P-P)	0.255	8.95*10^-3	7.35*10^-5	0.75	0.242–0.260
D2	305	Absorbance (p-o)	0.253	8.22*10^-3	8.23*10^-5	0.64	0.243–0.259
D2	287	Absorbance (p-o)	0.252	8.45*10^-3	8.25*10^-5	0.33	0.244–0.262
D2	296	Absorbance (p-o)	0.25	7.25*10^-3	7.25*10^-5	0.2	0.244–0.253
D2	262–276	Peak(P-P)	0.255	8.65*10^-3	8.25*10^-5	0.14	0.246–0.265
D2	276–297	Peak(P-P)	0.251	8.25*10^-3	7.25*10^-5	0.88	0.240–0.258
D2	297–318	Peak(P-P)	0.25	7.25*10^-3	8.59*10^-5	0.14	0.242–0.264
D2	262–318	Peak(P-P)	0.251	9.25*10^-3	9.12*10^-5	0.87	0.244–0.262
D3	278	Absorbance (p-o)	0.254	8.32*10^-3	9.02*10^-5	0.74	0.246–0.265
D3	296	Absorbance (p-o)	0.253	8.21*10^-3	8.25*10^-5	0.62	0.242–0.263
D3	311	Absorbance (p-o)	0.253	7.98*10^-3	8.75*10^-5	0.8	0.243–0.262
D3	267–287	Absorbance (p-o)	0.251	8.54*10^-3	8.85*10^-5	0.25	0.249–0.267
D3	287–306	Peak(P-P)	0.252	8.23*10^-3	7.65*10^-5	0.64	0.245–0.264
D3	306–317	Peak(P-P)	0.253	7.22*10^-3	8.15*10^-5	0.77	0.245–0.264
D3	267–317	Peak(P-P)	0.253	8.15*10^-3	9.14*10^-5	0.92	0.242–0.263
D4	270	Peak(P-P)	0.254	8.99*10^-3	9.45*10^-5	0.35	0.242–0.262
D4	287	Peak(P-P)	0.252	5.01*10^-3	5.45*10^-5	0.61	0.244–0.263

**Table 12:** Statistical evaluation of the elaborated method for norfloxacin (standard solution n = 10)

Derivative	λ (nm)	Technique	Regression equation	Correlation coefficient
D1	278	Absorbance (p-o)	y = 0.002x–0.0092	0.948
D1	300	Absorbance (p-o)	y = 0.0016x + 0.0103	0.997
D1	316–286	Peak(P-P)	y = 0.0359x–0.0199	0.941
D1	257–286	Peak(P-P)	y = 0.027x + 0.1501	0.990
D1	257–316	Peak(P-P)	y = 0.0134x–0.014	0.970
D2	310	Absorbance (p-o)	y = 0.0002x + 0.0006	0.979
D2	291	Absorbance (p-o)	y = 0.0004x–0.0014	0.974
D2	270	Absorbance (p-o)	y = 0.0002x + 0.0006	0.995
D2	254–279	Peak(P-P)	y = 0.0003x + 0.014	0.977
D2	279–298	Peak(P-P)	y = 0.0032x–0.015	0.985
D2	298–337	Peak(P-P)	y = 0.0042x + 0.0179	0.960
D2	254–337	Peak(P-P)	y = 0.0041x + 0.0163	0.962
D3	262	Absorbance (p-o)	y = 0.005x–0.002	0.994
D3	282	Absorbance (p-o)	y = 0.0003x + 0.0001	0.992
D3	311	Absorbance (p-o)	y = 0.0005x–0.0005	0.966
D3	297	Absorbance (p-o)	y = 0.0005x + 0.0002	0.985
D3	270–291	Peak(P-P)	y = − 0.0005x–0.0023	0.962
D3	253–270	Peak(P-P)	y = 0.0016x + 0.0003	0.984
D3	291–309	Peak(P-P)	y = 0.0008x + 0.0022	0.989
D3	309–337	Peak(P-P)	y = − 0.0001x–0.0005	0.997
D3	253–337	Peak(P-P)	y = 0.0006x + 0.0012	0.995
D4	267	Absorbance (p-o)	y = 0.027x + 0.1501	0.997
D4	258	Absorbance (p-o)	y = 0.0134x–0.014	0.951
D4	304	Absorbance (p-o)	y = 0.0002x + 0.0006	0.998
D4	321	Absorbance (p-o)	y = 0.0004x–0.0014	0.997
D4	291	Absorbance (p-o)	y = 0.0002x + 0.0006	0.985
D4	263–282	Peak(P-P)	y = 0.0003x + 0.014	0.998
D4	249–363	Peak(P-P)	y = 0.0032x-0.015	0.995
D4	297–310	Peak(P-P)	y = 0.0042x + 0.0179	0.998
D4	282–297	Peak(P-P)	y = 0.0041x + 0.0163	0.990
D4	310–319	Peak(P-P)	y = 0.005x-0.002	0.997
D4	249–319	Peak(P-P)	y = 0.0003x + 0.0001	0.989

**Table 13:** Statistical analysis of the determination of norfloxacin in tablets “Epinor 400 mg” (n = 10)

Derivative	λ (nm)	Technique	Content of norfloxacin (g)	Standard deviation	Variance	Correlation coefficient	Confidence interval (95%)
D1	278	Absorbance (p-o)	0.402	9.67*10^-3	9.05*10^-5	0.81	0.343–0.464
D1	300	Absorbance (p-o)	0.405	6.20*10^-3	9.66*10^-5	0.41	0.395–0.421
D1	316–286	Peak(P-P)	0.401	8.25*10^-3	9.23*10^-5	0.65	0.380–0.435
D1	257–286	Peak(P-P)	0.401	7.62*10^-3	9.56*10^-5	0.58	0.365–0.475
D1	257–316	Peak(P-P)	0.403	8.52*10^-3	7.25*10^-5	0.2	0.412–4.265
D2	310	Absorbance (p-o)	0.402	9.24*10^-3	8.21*10^-5	0.26	0.389–4.005
D2	291	Absorbance (p-o)	0.402	9.87*10^-3	7.75*10^-5	0.52	0.392–0.421
D2	270	Absorbance (p-o)	0.407	8.26*10^-3	7.95*10^-5	0.8	0.392–0.410
D2	254–279	Peak(P-P)	0.403	9.65*10^-3	9.15*10^-5	0.75	0.385–0.402
D2	279–298	Peak(P-P)	0.404	9.66*10^-3	8.89*10^-5	0.2	0.396–0.419
D2	298–337	Peak(P-P)	0.402	7.25*10^-3	7.12*10^-5	0.69	0.400–0.415
D2	254–337	Peak(P-P)	0.403	8.32*10^-3	7.95*10^-5	0.7	0.394–0.411
D3	262	Absorbance (p-o)	0.404	9.21*10^-3	9.14*10^-5	0.31	0.410–0.425
D3	282	Absorbance (p-o)	0.402	8.22*10^-3	9.05*10^-5	0.8	0.408–0.417
D3	311	Absorbance (p-o)	0.406	7.45*10^-3	9.14*10^-5	0.95	0.384–0.414
D3	297	Absorbance (p-o)	0.404	9.12*10^-3	7.45*10^-5	0.62	0.397–0.408
D3	270–291	Peak(P-P)	0.403	8.98*10^-3	9.32*10^-5	0.31	0.380–0.405
D3	253–270	Peak(P-P)	0.404	8.14*10^-3	9.65*10^-5	0.95	0.393–0.415
D3	291–309	Peak(P-P)	0.405	8.25*10^-3	8.32*10^-5	0.45	0.382–0.407
D3	309–337	Peak(P-P)	0.402	9.25*10^-3	9.68*10^-5	0.9	0.382–0.400
D3	253–337	Peak(P-P)	0.403	9.25*10^-3	9.68*10^-5	0.2	0.373–0.404
D4	267	Absorbance (p-o)	0.404	5.87*10^-3	5.21*10^-5	0.65	0.384–0.421
D4	258	Absorbance (p-o)	0.404	5.24*10^-3	5.54*10^-5	0.65	0.392–0.424
D4	304	Absorbance (p-o)	0.403	4.02*10^-3	4.70*10^-5	0.6	0.374–0.416
D4	321	Absorbance (p-o)	0.404	3.25*10^-3	3.25*10^-5	0.62	0.437–0.264
D4	292	Absorbance (p-o)	0.403	5.97*10^-3	5.35*10^-5	0.52	0.403–0.410
D4	263–282	Peak(P-P)	0.405	3.20*10^-3	3.14*10^-5	0.65	0.402–0.422
D4	249–363	Peak(P-P)	0.403	3.63*10^-3	3.25*10^-5	0.61	0.403–0.464

**Table 14:** Statistical evaluation of the elaborated method for moxifloxacin (standard solution n = 10)

Derivative	λ (nm)	Technique	Regression equation	Correlation coefficient
D1	281	Absorbance (p-o)	y = 0.0013x + 0.0241	0.972
D1	304	Absorbance (p-o)	y = − 0.0013x–0.0244	0.972
D1	292–326	Peak(P-P)	y = − 0.0222x–0.4592	0.979
D1	262–292	Peak(P-P)	y = 0.0206x + 0.3726	0.960
D1	262–326	Peak(P-P)	y = − 0.0048x–0.0615	0.994
D2	311	Absorbance (p-o)	y = 0.0001x + 0.0024	0.964
D2	294	Absorbance (p-o)	y = − 0.0002x–0.004	0.960
D2	271	Absorbance (p-o)	y = 0.00009x + 0.00173	0.978
D2	262–278	Peak(P-P)	y = 0.0005x + 0.0091	0.948
D2	278–304	Peak(P-P)	y = − 0.0035x–0.0654	0.948
D2	304–331	Peak(P-P)	y = 0.0012x + 0.0261	0.942
D2	262–331	Peak(P-P)	y = − 0.0017x–0.0322	0.967
D3	287	Absorbance (p-o)	y = − 0.00002x–0.00041	0.942
D3	302	Absorbance (p-o)	y = 0.00004x + 0.00073	0.946
D3	315	Absorbance (p-o)	y = − 0.00001x–0.00026	0.963
D3	263–295	Peak(P-P)	y = − 0.0005x–0.0093	0.964
D3	295–309	Peak(P-P)	y = 0.0008x + 0.0022	0.948
D3	309–332	Peak(P-P)	y = − 0.0002x–0.0031	0.970
D3	263–332	Peak(P-P)	y = − 0.0003x–0.0065	0.953
D4	271	Absorbance (p-o)	y = − 0.000001x–0.000027	0.998
D4	277	Absorbance (p-o)	y = − 0.000002x–0.000050	0.998
D4	294	Absorbance (p-o)	y = 0.000008x + 0.000174	0.956
D4	307	Absorbance (p-o)	y = − 0.00001x–0.00016	0.940
D4	320	Absorbance (p-o)	y = 0.000001x + 0.000028	0.956
D4	266–273	Peak(P-P)	y = 0.000001x + 0.000009	0.990
D4	273–282	Peak(P-P)	y = − 0.000009x–0.000111	0.989
D4	282–302	Peak(P-P)	y = 0.00008x + 0.00160	0.957
D4	302–313	Peak(P-P)	y = − 0.00005x–0.00097	0.956
D4	313–332	Peak(P-P)	y = 0.00004x + 0.00028	0.968

**Table 15:** Statistical evaluation of moxifloxacin determination in “Delmoxa 400” tablets (n = 10)

Derivative	λ (nm)	Technique	Content of moxifloxacin (g)	Standard deviation	Variance	Correlation coefficient	Confidence interval (95%)
D1	281	Absorbance (p-o)	0.4	8.67*10^-3	7.45*10^-5	0.31	0.4025–0.426
D1	304	Absorbance (p-o)	0.401	9.68*10^-3	9.91*10^-5	0.25	0.395–0.405
D1	292–326	Peak(P-P)	0.4	8.85*10^-3	8.65*10^-5	0.53	0.393–0.428
D1	262–292	Peak(P-P)	0.4	7.78*10^-3	9.36*10^-5	0.52	0.387–0.405
D1	262–326	Peak(P-P)	0.4	9.56*10^-3	9.52*10^-5	0.9	0.394–0.411
D2	311	Absorbance (p-o)	0.401	7.65*10^-3	9.85*10^-5	0.85	0.405—0.419
D2	294	Absorbance (p-o)	0.4	5.32*10^-3	5.65*10^-5	0.91	0.401–0.429
D2	271	Absorbance (p-o)	0.4	9.65*10^-3	9.55*10^-5	0.66	0.389–0.415
D2	262–278	Peak(P-P)	0.4	7.65*10^-3	8.65*10^-5	0.8	0.390–0.425
D2	278–304	Peak(P-P)	0.401	9.89*10^-3	9.80*10^-5	0.52	0.407–0.418
D2	304–331	Peak(P-P)	0.401	7.95*10^-3	8.65*10^-5	0.6	0.401–0.426
D2	262–331	Peak(P-P)	0.4	9.45*10^-3	8.25*10^-5	0.41	0.403–0.424
D3	287	Absorbance (p-o)	0.401	7.69*10^-3	9.65*10^-5	0.32	0.388—0.425
D3	302	Absorbance (p-o)	0.401	5.32*10^-3	4.65*10^-5	0.45	0.381–0.410
D3	315	Absorbance (p-o)	0.4	9.55*10^-3	9.65*10^-5	0.71	0.394–0.405
D3	263–295	Peak(P-P)	0.401	7.98*10^-3	9.59*10^-5	0.46	0.406–0.420
D3	295–309	Peak(P-P)	0.401	8.80*10^-3	9.51*10^-5	0.25	0.405–0.420
D3	309–332	Peak(P-P)	0.401	9.78*10^-3	8.45*10^-5	0.17	0.400–0.415
D3	263–332	Peak(P-P)	0.4	8.65*10^-3	9.65*10^-5	0.81	0.408–0.415
D4	271	Absorbance (p-o)	0.401	9.54*10^-3	7.98*10^-5	0.15	0.390–0.420
D4	277	Absorbance (p-o)	0.401	8.90*10^-3	9.25*10^-5	0.64	0.395–0.427
D4	294	Absorbance (p-o)	0.401	7.45*10^-3	7.77*10^-5	0.92	0.389–0.423
D4	307	Absorbance (p-o)	0.401	8.65*10^-3	8.77*10^-5	0.25	0.397–0.402
D4	320	Absorbance (p-o)	0.401	8.77*10^-3	7.69*10^-5	0.15	0.398–0.412
D4	266–273	Peak(P-P)	0.4	9.45*10^-3	9.45*10^-5	0.22	0.371–0.420
D4	273–282	Peak(P-P)	0.4	8.22*10^-3	9.56*10^-5	0.36	0.399–0.417
D4	282–302	Peak(P-P)	0.4	9.46*10^-3	9.33*10^-5	0.55	0.387–0.408
D4	302–313	Peak(P-P)	0.402	9.52*10^-3	9.41*10^-5	0.47	0.400–0.415
D4	313–332	Peak(P-P)	0.4	8.74*10^-3	8.65*10^-5	0.16	0.398–0.401

Potency assay tests of levofloxacin were performed by the proposed method. According to USP 29 [38], levofloxacin tablets must contain 95–105% of the labeled amount of drug. The brand products met the standard criteria with the new analytical method (Table 11). For Levofloxacin in Levonex tablets, the "peak-zero" technique for the fourth derivative spectra at 287.0 nm yielded the best results (SD—5.01*10^-3, RSD—0.61%).

Potency assay tests of norfloxacin were performed by the proposed method. According to USP 29 [38], norfloxacin tablets must contain 95–105% of the labeled amount of drug. The brand products met the standard criteria with the new analytical method (Table 13). For Norfloxacin in Epinor 400 mg tablets showed optimal results with the "peak-zero" and "peak-peak" techniques for the fourth derivative spectra at all examined wavelengths (RSD about 0.65%).

Potency assay tests of moxifloxacin were performed by the proposed method. According to USP 29 [38], Moxifloxacin tablets must contain 95–105% of the labeled amount of drug. The brand products met the standard criteria with the new analytical method (Table 15). For Moxifloxacin in Delmoxa tablets demonstrated superior outcomes with the "peak-zero" technique for the second derivative spectrum at wavelength 294 nm (SD—5.3210^-3, RSD—0.91%) and the third derivative spectrum at wavelength 302 nm (SD—5.3210^-3, RSD—0.45%).

The table presents the statistical evaluation of the determination of Moxifloxacin content in "Delmoxa 400" tablets using various derivatives and techniques.

These results are provided to assess the accuracy and efficiency of the proposed method used for the analysis of Moxifloxacin in “Delmoxa 400” tablets.

## Discussion

In summary, the proposed analytical method proves to be a highly effective tool for the routine quality control analysis of Levofloxacin, Norfloxacin, and Moxifloxacin in both pure and pharmaceutical samples employing. First-, second-, third-, and fourth-order UV derivative spectroscopy, offers a straightforward, quick, sensitive, and direct approach for determining the analyzed drugs. The method offers accuracy, precision, selectivity, and ease of use, making it a valuable addition to the analytical toolbox.

Utilizing the Lambda 15 spectrophotometric, spectra derivatives can be stored in the computer memory, allowing the retrieval of derivative values at any marked point on the recorded spectrum. Two graphical techniques, namely “peak-zero” and “peak-peak,” were employed for determining spectra derivatives.

In the “peak-peak” technique, amplitude measurements (from maximum to minimum) were conducted, while the baseline-to-peak technique involved measurements from the maximum to the zero line or from the minimum to the zero line.

Linear equations derived from regression analysis for Levofloxacin, Norfloxacin, and Moxifloxacin are presented in Tables 10, 12, and 14, respectively. Statistical evaluation of the results for the determination of fluoroquinolones in pharmaceutical dosage tablets is illustrated in Tables 11, 13, and 15.

## Conclusions

The proposed approach demonstrates sensitivity, accuracy, simplicity, precision, speed, and economy. The results showed an outstanding mean recovery (99.20–100.08%) and a good correlation value (R2 = 0.999).The recommended derivative spectrophotometry method was successfully validated in accordance with ICH Q2B criteria by using UV spectral data that was produced from the analysis of chemically deteriorated samples using the recommended methodology. In physical–chemical research, derivative spectrophotometry proves to be an extremely useful tool as it allows for the non-invasive extraction of information from the fundamental spectrum. Recovery investigations support the great accuracy of these techniques even further. The method is found to be precise and accurate, with a linear response within the given range and lower LOD and LOQ values where acceptable, making it suitable for routine quality control analysis for levofloxacin, Norfloxacin, and Moxifloxacin in both pure form and pharmaceutical samples.

### Supplementary Information

**Additional file 1.** Examples Spectra Used In The Research.

## Data Availability

The datasets during and/or analysed during the current study available from the corresponding author on reasonable request.
