# Supplementary material for: Determination of levofloxacin, norfloxacin, and moxifloxacin in pharmaceutical dosage form or individually using derivative UV spectrophotometry
Source: BMC Chem. 2024 Jun 14;18(1):115. doi: 10.1186/s13065-024-01193-4 (PMC11179347; doi:10.1186/s13065-024-01193-4)
Supplement: Supplementary file 1 — Additional file 1. Examples Spectra Used In The Research. [file 13065_2024_1193_MOESM1_ESM.docx]

Examples Spectra Used In The Research


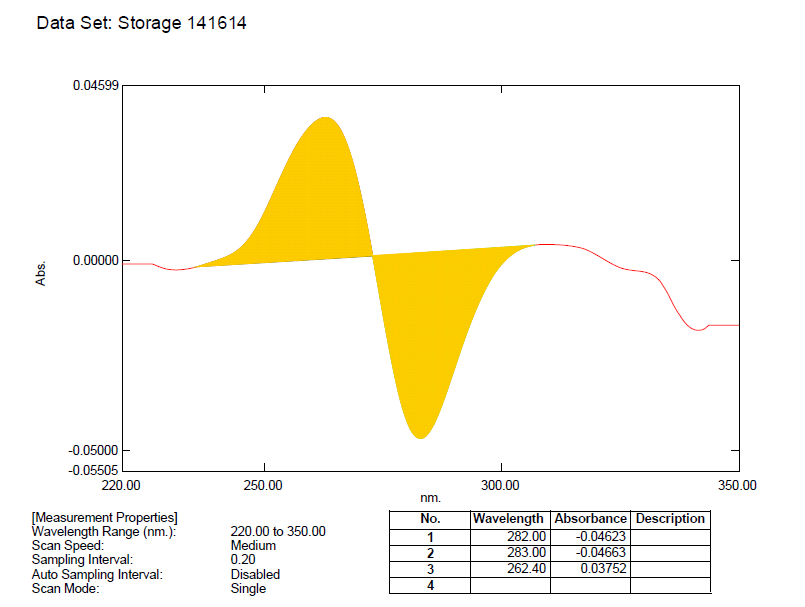


Figure 1 showed the 1^st^ derivative “P to O”.


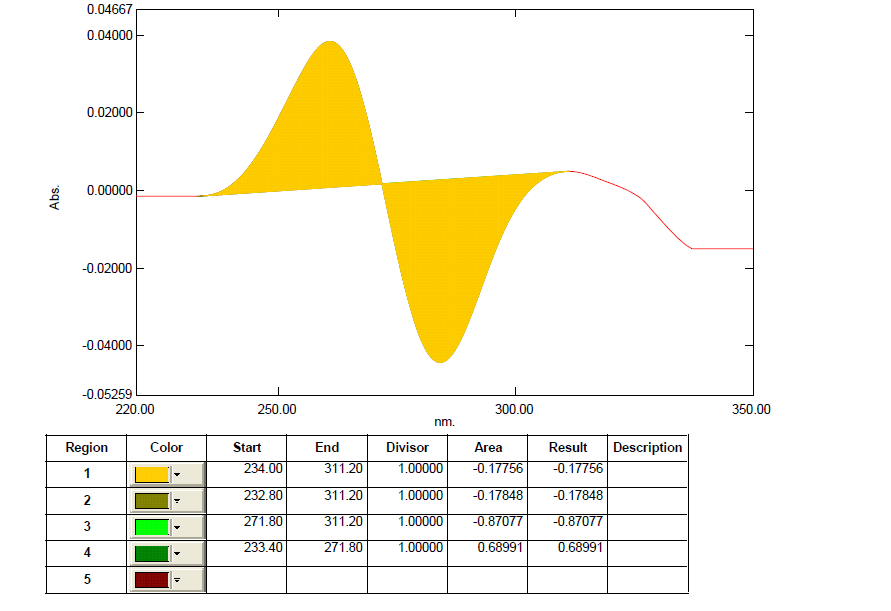


Figure 2 showed the 1^st^ derivative “P to P”.


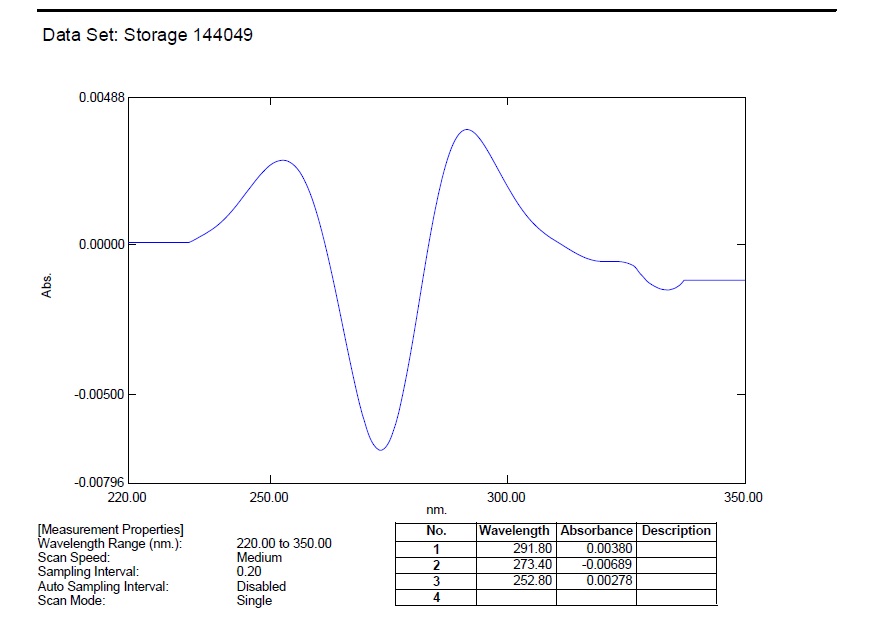


Figure 3 showed the 2^nd^ derivative “P to O”.


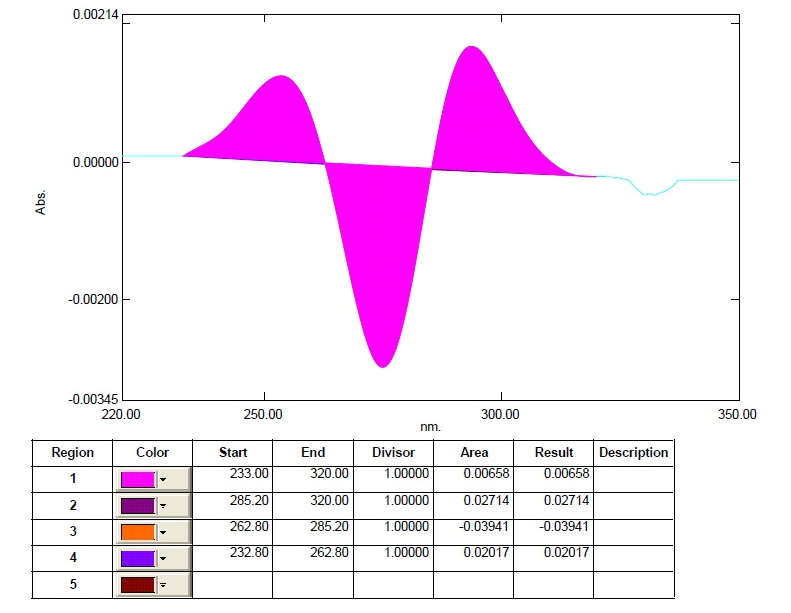


Figure 4 showed the 2^nd^ derivative “P to P”.


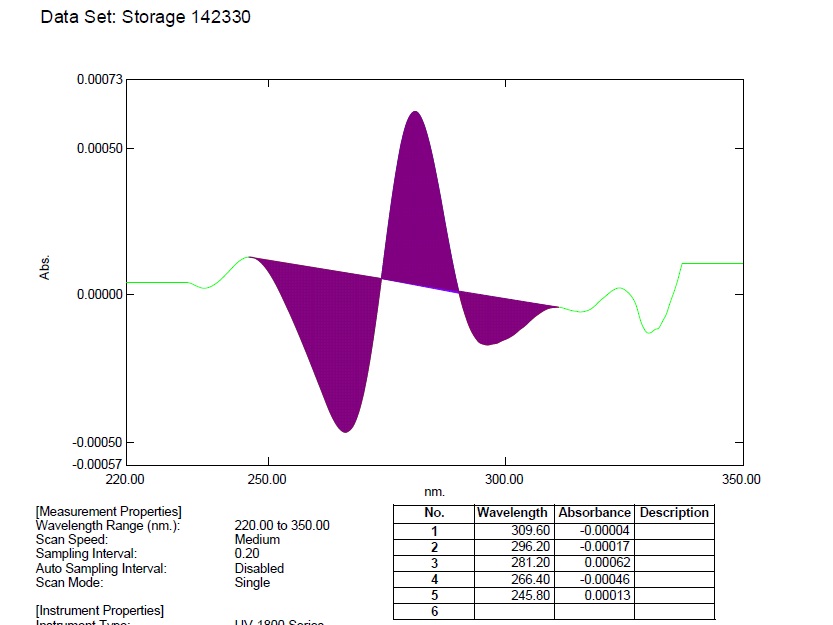


Figure 5 showed the 3^rd^ derivative “P to O”.


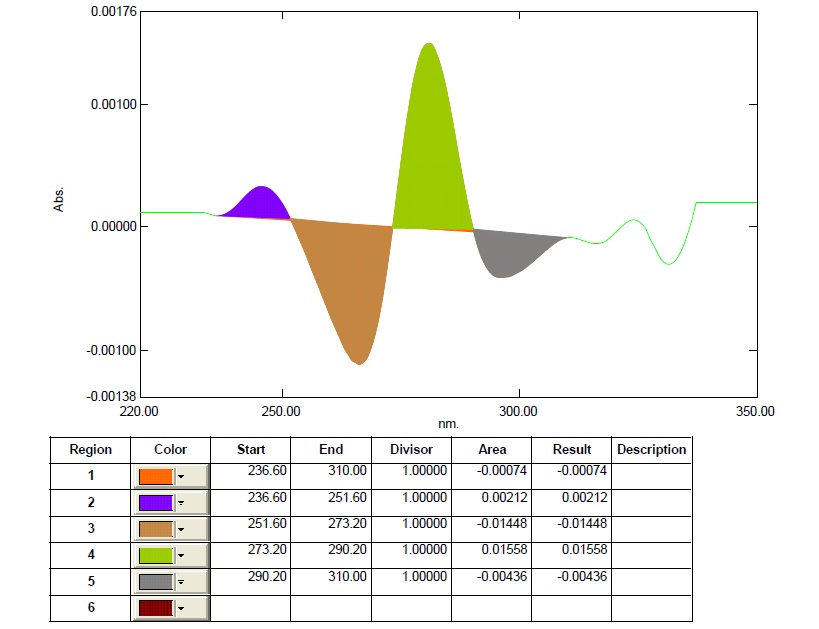


Figure 6 showed the 3^rd^ derivative “P to P”.


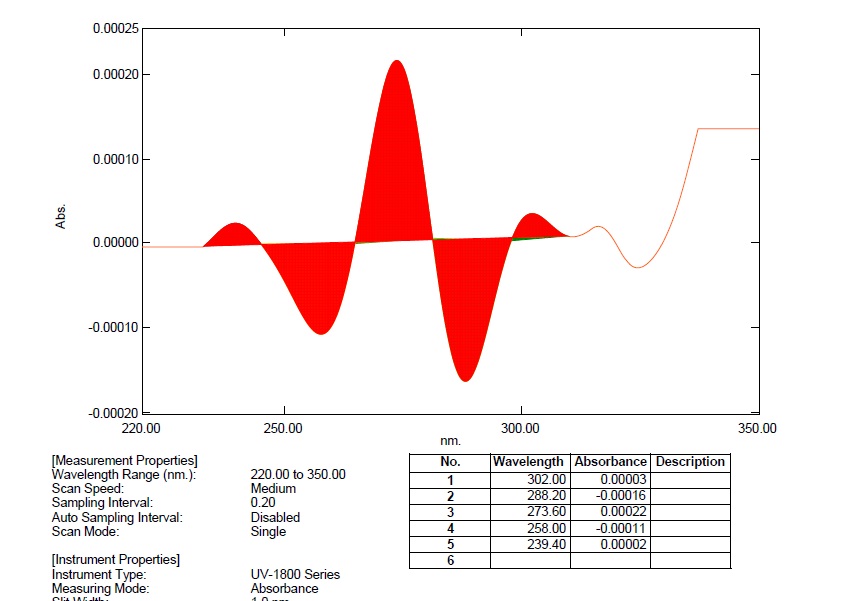


Figure 7 showed the 4 ^th^ derivative “P to O”.


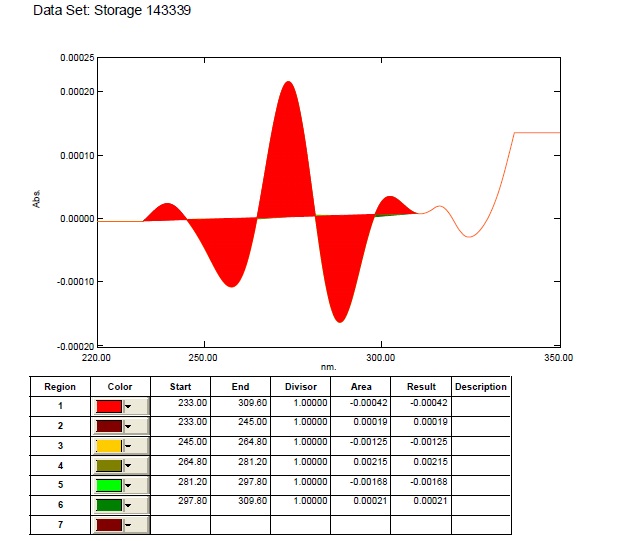


Figure 8 showed the 4 ^th^ derivative “P to P”.


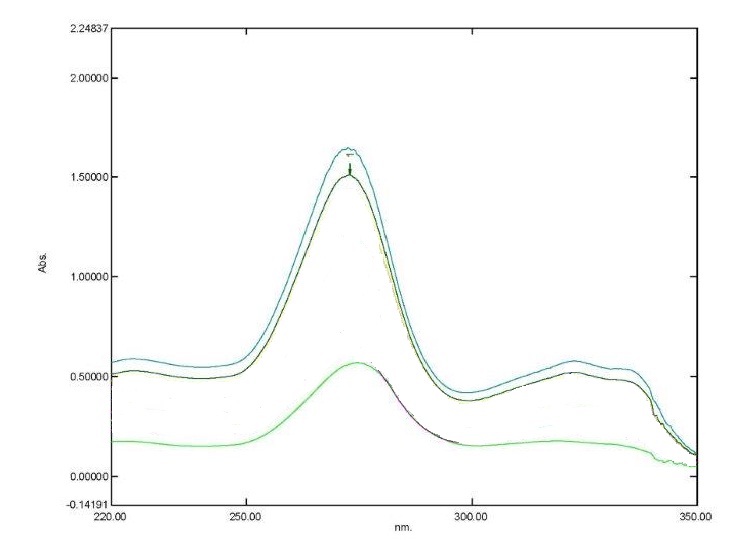


Figure 9 Overlain UV spectra of pH effect on norfloxacin
